# Predictors of acculturation attitude of international students in China

**DOI:** 10.1371/journal.pone.0260616

**Published:** 2021-11-30

**Authors:** Man Luo, Xiaofang Zhang, Fang Peng, Jing Zhao, Haijun Deng

**Affiliations:** Department of Public Health, Yangtze University, Jingzhou, Hubei, China; Rzeszow University of Technology: Politechnika Rzeszowska im Ignacego Lukasiewicza, POLAND

## Abstract

This study investigates international students’ acculturation attitude preference and the influence factors (i.e., gender, duration in China, host and English proficiency, adult attachment style and social ties) on each acculturation attitude (i.e, integration, assimilation, separation and marginalization attitude) in China. A group of 229 international students within China responded online. Results from cluster analysis based on process model of acculturation revealed that, integration was the most prevalent acculturation attitude of international students in China. Additionally, logistic regressions analysis indicated that duration in China was negatively associated with adoption of separation and marginalization attitude also higher level of English proficiency predicted the preference of marginalization attitude. However, Chinese proficiency was positively associated with integration attitude adoption, host ties were positively and significantly associated with adoption of assimilation and integration attitude, but negatively associated with the usage of marginalization attitude. Lastly, avoidance attachment was found negatively associating with the integration attitude. This study provides valuable insights for international student education policymakers to assist international students in order to adapt to a new environment quickly and effectively.

## Introduction

According to the data of Ministry of Education of the People’s Republic of China, there were 492,185 foreign students from 196 countries studying in China in 2018 [1]. Different natural environments, social cultures brought about various difficulties for foreign students, if they lack enough response measures to the various difficulties, cross-cultural adaptation will result in failure. The failure of cross-cultural adaptation will lead to academic failure and even serious mental illness on international students, such as anxiety, depression and suicide ideation [2–4]. Therefore, there is an urgent need to understand what problems they face and how they adopt to China during acculturation process.

Actually, numerous studies have been carried out in different contexts in order to better understand individuals’ acculturation process in a new environment [5, 6]. Those researches are reflected at the group and individual level. The group level pays more attention on the changes of the country’s intellectual capability, financial stability, and political organization [7, 8], whereas the individual level mainly focus on influencing factors on psychological and sociocultural adaptations, such as age, gender, country of origin, education level [2], proficiency in English and the host language, and length of residence [5], perceived stereotype image [9], Social Support, friendship network [4, 10], and so on.

Past researches have established that acculturation strategies have effect on individuals’ cross-cultural adaptation [11–13]. Jia-Yan Pan [11] found that marginalization or assimilation strategy significantly predicted negative affect on Chinese international students whom studied in Melbourne. Ting Kin Ng [12] revealed that the effects of integration and marginalization attitude on psychological adaptation significantly mediated sociocultural adaptation. As an important aspect in the cross-cultural adaptation process, it is necessary to understand the influencing factors of acculturation attitude.

A substantial body of research has led to the understanding that the acculturation process was related to individual’s demographic and social characteristics [14–16]. Thus, individual’s demographic and social characteristics should be examined when studying an international students’ acculturation attitude. However, little is currently known about these relationships in the context of China. For this reason, the present study focuses and consequently tests the effect of some sociodemographic variables and social characteristics on acculturation attitudes among international students in China. Several predictors identified and discussed in detail in the subsequent sections are: sociodemographic variables, social ties and attachment style.

### Acculturation attitudes

Berry and his associates [17] proposed a bidimensional model of acculturation, which provides a theoretical framework for the study of acculturation attitudes: acculturating individuals’ heritage culture maintenance(to what extent the heritage culture should be maintained) and the local mainstream culture adoption(to what extent the host culture should be adopt). According to the answer of those two questions, four acculturation attitudes were yield. (i)Integration, in which individuals maintain their heritage culture and also absorb the local mainstream culture; (ii) Assimilation, in which individuals reject their heritage culture and completely absorb the local mainstream culture; (iii)Separation, in which individuals strongly retain their heritage culture but reject the local mainstream culture; and (iv)Marginalization, in which individuals lose their heritage culture without establishing ties with the local mainstream culture.

### Effect of social ties on acculturation attitudes

Bochner, McLeod, and Lin [18] were the first to study international students’ friendship forms. Subsequently, friendship forms of international students have been widely investigated by researchers [10, 19, 20]. Three distinct social ties were founded in the host country: the primary was co-national ties (friendships with compatriots); the secondary was host-national ties (friendships with the host country people); and the tertiary was international ties (friendships with people from different countries except host country).

The contact hypothesis and its extensions [21] shed some light on the relationship between intergroup(s) contact and acculturation attitude. However, the bulk of the inter group contact literature focuses on how contact affects the majority’s attitudes [22], a much smaller literature examining possible effects on minority individuals. Lebedeva N, Tatarko A, Berry JW [23] investigated the relationship between acculturation strategies and social support in a sample of migrants in Moscow, and founded that inter group contact was a predictor of a high level of identification with the host culture. In a study by Szabó Á, Z. and his/her colleague(s) [20], the results align with previous findings that the host culture orientation was associated with frequent contact with locals, whereas home cultural orientation was correlated with frequent contacts with co-nationals. On the other hand, in a study by Berger [24] with exchange students in Spain and Germany, no significant association between separation acculturation strategy and co-national social ties were found. In the present study, we want to find the effect of social ties on acculturation attitudes in terms of international students in China.

### Effect of attachment style on acculturation attitudes

Brennan, Clark, &Shaver [25] have proposed that adult attachment consists of two dimensions, labeled anxiety and avoidance. Attachment anxiety involves an excessive need for approval from others and fear of interpersonal rejection, whereas attachment avoidance involves an excessive need for personal independence and thus avoiding intimacy. According to the answer of those two dimensions, four types attachment styles were founded. The secure attachment style is characterized by low levels of anxiety and avoidance. On the contrary, the fearful attachment indicates high level of anxiety and avoidance. The preoccupied attachment style indicates low levels of avoidance but high levels of anxiety. In contrast, individuals with a dismissing attachment style have high levels of avoidance but low levels of anxiety.

As attachment theory refers to interaction with others in new environment, there is reason to believe association between attachment style and acculturation attitude. Actually, prior research have provided some evidences for the relationship between attachment style and acculturation attitudes. Jacomijn Hofstra [26] founded that secure attachment style was a significant positive predictor of the integration, assimilation and marginalization strategies. Furthermore, the dismissive and secure attachment styles were significant positive predictors of separation strategy. Wang, C.D. and B. Mallinckrodt [27] founded that attachment anxiety was negatively associated with students’ acculturation to U.S. culture. Ferenczi, N. [28] revealed that secure-preoccupied nation attachment was a significant predictor of increased heritage culture identification for participants residing in their country of birth, while dismissive nation attachment was a significant predictor of decreased heritage culture identification for international migrants. However, little is currently known about these relationships in the context of international students.

### Effects of demographic variables on acculturation attitude

Variables such as age, gender, Place of origin, marital status and religious affiliation in the host country are considered related to the acculturation attitude. For instance, girls were more likely to adopt integration strategy than boys in Olga Pisarenko’s study of Russian adolescents in Latvia [29]. While some other studies found that there is no relationship between gender and acculturation attitude [12]. Needham, B.L., et al [30] founded that immigrants with no religious affiliation were likely adopting assimilation than separation. It had been founded that good financial conditions were positive correlated with social network building in the host country. Therefore, the higher level of financial conditions, the higher odds of adopting either integration or assimilation attitude over separation attitude [30]. Married immigrants or international students need to invest much of their time in being with their spouses, which, in turn, decrease opportunities for social interactions and thus lead to adoption of segregation attitude [31]. Furthermore, there were some other studies that revealed that those variables cannot be used while predicting acculturation attitude [12, 32, 33].

Language is a useful tool for foreigners to communicate and socialize with host people, which is important for broadening social network and resources. Kashima & Loh [33] found that People with higher host country’s language proficiency have a higher degree of identification with the host culture because of intercultural interactions. Needham, B.L., et al [30] revealed that those who were fluent in English had higher odds of adopting assimilation or integration strategies than the separation strategy, but there is no statistically significant relatedness for marginalization and language proficiency.

Length of residence in the host country is widely reported to influence the acculturation attitude. Studies of multicultural students in Canada showed that the longer their length of residence in Canada, the greater mainstream cultural adoption, and the lower heritage cultural maintenance [34]. Szabó Ágnes and colleagues [20] investigated acculturation attitudes in a sample of international students in the capital city of Hungary at formal and informal context and found that length of residence in the host country was negatively associated with host country orientation but no correlation with home country orientation. Similar yet different result was reported for immigrants. Professional Chinese immigrants who resided in the Australian for a longer period have enough time to gain the experience in the host society, promoting the will to participate in the mainstream society, so the length of residence in the host country were positively associated with assimilation attitude [35].

## The present study

In the present study, we aim to find influence factors of acculturation strategies in international students, which can provide valuable information for education policymakers in China. Based on above the literature review, we hypothesize that some demographic variables, attachment style, social ties in China may influence international students’ acculturation attitude in the host country. To better understand their potential relationships, we formulate the following hypotheses:

Hypothesis 1: Duration in China will be positively related to integration and assimilation, and negatively related to separation and marginalization.
Hypothesis 2: Language proficiency will be positively related to integration and assimilation, and negatively related to separation and marginalization.
Hypothesis 3: Avoidance will be positively related to integration and assimilation, and anxiety positively related to separation and marginalization.
Hypothesis 4: Host ties will be positively related to integration and assimilation, and negatively related to separation and marginalization. Co-national ties will be positively related to integration and separation, international ties will be positively related to integration.

## Method

### Participants

The survey received approval by Medical ethics committee of Yangtze university and conducted between November 2019 and January 2020. Written consent was obtained from the participants at the beginning of questionnaire and the consent was informed. All the participants were over 18 years old.

International students were recruited from Yangtze university in Hubei province of China. Yangtze University was selected by convenient sampling method among the general international students studying in China, as it is one of the famous universities with the largest number of nationalities, including students of Pakistan, Zambia, Ethiopia, Ghana, Indonesia, Canada, France and so on. International students of Yangtze University were selected using stratified sampling method with duration in China and nationality as stratified variables. Trained data collectors approached participants directly with the assistance of the Yangtze University staff and international student leaders. An anonymous electronic questionnaire was used for the data collection. Participants individually complete the survey on their cellphones. Prior to the survey, all participants were informed to answer each statement as honestly as possible, because the survey was being conducted anonymous and strictly confidential manner. Out of the 235 participants approached in this way, 229 (97%) participants from 20 countries completed the survey, consisting of 94(41.0%) Pakistanis,13(5.68%) Nepalese, 1(0.44%)Thai, 9(3.93%)Indonesians, 22(9.61%)Zimbabwean, 16(6.99%) Ghanaians, 18(7.86%) Tanzanians, 2(0.87%) Ugandans, 22(9.61%)Zambians, 1(0.44%)Gabonese, 13(5.68%)Rwandans, 5(2.18%)Ethiopians, 1(0.44%)Chadian, 1(0.44%)South African, 1(0.44%)Papua New Guinea, 2(0.87%)Congolese, 1(0.44%)Namibians, 3(1.31)Canadian, 2(0.87%)French, 2(0.87%)Britisher. This sample composition is fairly representative for international students in China. 123(53.7%) of participants were male, and 106(46.3%) of participants were female, the average age of male and female was 22.12 (SD = 2.20) and 21.25 (SD = 1.39) respectively.

This study examined international students’ acculturation attitude from the perspective of demographic factors, attachment style and social support. Cluster analysis was conducted to divide acculturation strategies into integration, separation, assimilation and marginalization. *χ*2 test and One-Way ANOVA test was used to profiled the demographic factors of international students with regard to their acculturation attitudes. Four binary logistic regression analysis were used to find out what specific predictors such as duration in China, language proficiency, attachment style and social support could impact participants’ integration, assimilation, separation, and marginalization attitudes respectively.

### Measures

#### Demographics

Participants’ age, gender, nationality, current marital status, time spent in the China, English language proficiency and Chinese language proficiency were included in the questionnaire. Self-report was used to assess students’ English and Chinese proficiency instead of their scores on TOEFL/ IELTS and HSK. Because we believe spoken proficiency plays a crucial role in the acculturation process. Example of English proficiency “How good do you think your overall English language ability is?” followed by five ordinal choices: 1: very poor,2: poor, 3: moderate, 4: good and 5: very good.

#### Social ties

The modified Multi-Dimensional Support Scale [36] was used to measure perceived social support from different sources. In the current study, this scale was used to assess social support from (a) host friends (Chinese friends), (b)Co-national friends (same nationality friends), and (c) international friends (different nationality friends except Chinese). Each source of social support is measured with 4 items on a 4-point Likert scale (1 = never, 4 = always). In this study, Cronbach’s a values for these subscales were 0.85,0.84 and 0.84, respectively.

#### Adult attachment

This was assessed by Meifen Wei and Daniel W. Russell’s [37] ECR Scale-Short Form. This instrument consists of 12 items evaluating two dimensions of attachment: Avoidance and Anxiety, using a seven-point scale ranging from 1 (strongly disagree) to 7 (strongly agree). Avoidance consists of six items reflecting avoidance in relationships, such as hard to establish close relationship with important people or trust them (“I want to get close to my partner, but I keep pulling back”). Anxiety consists of six items reflecting anxiety in relationships, such as fear of being abandoned and not being loved (“I worry that romantic partners won’t care about me as much as I care about them”). Cronbach’s alphas for Avoidance and Anxiety subscales were .54 and .54, respectively.

#### Acculturation strategy

The measure of acculturation strategy in this study was adapted from ‘Adopt and Keep’ scale developed by Swaidan et al. [38]. The scale consisted of 10 items evaluating two dimensions of strategy: ‘adopt’ and ‘keep’, using a 5-point Likert-type scale ranging from ‘1’ (strongly disagree) to ‘5’ (strongly agree).The ‘adopt’ sub-scale measured international students’ tendency to adjust to the host country’s cultural, (e.g., “I should behave in accordance with Chinese culture”),while the ‘keep’ sub-scale measured their tendency to attach to their own original country’s culture (e.g., “I should remain attached to my original culture”). Cronbach’s alphas for ‘adopt’ and ‘keep’ subscales and the total scale were .84 and .90, respectively.

## Results

### Demographic characteristic of participants

Descriptive statistics for the analysis of international students’ acculturation attitude with regard to demographic characteristic were presented (Table 1). Among those variables, only gender is founded associated with acculturation strategies (*χ*^2^ = 8.434, *P* = 0.038), for which female are more preferred to integration than male, nevertheless male are more preferred to marginalization than female. Consider presenting general demographic data too like not only objective oriented ones.

**Table 1:** Demographic characteristics of study participants.

Variables	Categories	Separation n(%)	Marginalization n(%)	Assimilation n(%)	Integration n(%)	*χ*2	*P*
Gender	Male	32(26.0)	17(13.8)	20(16.3)	54(43.9)	8.434	0.038*
	Female	21(19.8)	7(6.6)	12(11.3)	66(62.3)		
Place of origin	Asia	33(28.2)	10(8.6)	21(17.9)	53(45.3)	11.494	0.074
	Africa	20(19.0)	12(11.4)	10(9.6)	63(60.0)		
	Others	0(0.0)	2(28.6)	1(14.3)	4(57.1)		
Marital status	Unmarried	51(22.8)	23(10.3)	32(14.3)	118(52.7)	1.934	0.586
	Married	2(40.0)	1(20.0)	0(0.0)	2(40.0)		
Religious belief	Christian	20(20.6)	12(12.4)	11(11.3)	54(55.7)	5.258	0.511
	Islam	26(24.1)	12(11.1)	16(14.8)	54(50.0)		
	Other	7(29.2)	0(0.0)	5(20.8)	12(50.0)		
Duration in China	1-12months	5(12.5)	7(17.5)	7(17.5)	21(52.5)	15.595	0.076
	13-24months	4(10.0)	5(12.5)	8(20.0)	23(57.5)		
	25-36months	21(34.4)	6(9.8)	7(11.5)	27(44.3)		
	>36 months	23(26.1)	6(6.8)	10(11.4)	49(55.7)		

*P < .05,

**P < .01,

***P < .005.

### Acculturation strategy classification

Firstly, cluster analysis was conducted linking these two acculturation strategies dimensions. In line with previous studies [35, 39], the produced clusters were divided in advance into four according to Berry’s theoretical assumptions, namely integration, marginalization, assimilation, and separation strategies. The results of the cluster analysis are presented in Table 2 and show that 52.4% of international students preferred to integration strategy, followed by separation (23.1%), assimilation (14.0%) and marginalization (10.5%). Then One-Way ANOVA analysis was performed to find whether there is differences between adjustment scores, attachment scores and acculturation strategies. As shown in Table 2, there is statistically significant differences between adjustment scores, attachment scores and acculturation strategies, as F_0.05,(3,225)_ = 176.821, F_0.05,(3,225)_ = 244.131, respectively, *P*<0.05.

**Table 2:** Cluster analysis of acculturation strategy.

Acculturation attitude	M(SD)					F
	Separation (N = 53)	Assimilation (32)	Integration (N = 120)	Marginalization (24)	Total (229)	
Adjustment	2.80(0.47)	4.04(0.36)	4.15(0.59)	1.89(0.50)	3.58(0.96)	176.821***
Attachment	3.41(0.64)	2.94(0.56)	4.56(0.43)	1.84(0.51)	3.79(1.05)	244.131***
Percentage(%)	23.1	14.0	52.4	10.5	100	

*P < .05,

**P < .01,

***P < .005.

### Predictors of separation

A binary logistic regression analysis was used to predict the chance that the international student would adopt separation. The Hosmer and Lemeshow test showed that the full model was valuable, with χ2 = 9.275, df = 8, p>0.05. The overall correct percentage was 76%. The full model explained 14.1% (Nagelkerke’s R2) of variance in the separation attitude of international students, with duration in China as the significant variable. As shown in Table 3, duration in China negatively influence separation attitude of international students. The odds ratio for duration in China was 0.718, indicating that holding all other variables constant in the model, with duration in China increase one year, the participant were 0.718 times less likely to adopted the separation attitude.

**Table 3:** Logistic regression predicting likelihood of adopting separation attitude.

	B	S.E	Wald	df	P	Exp(B)	95% CI for OR	
							lower	upper
Gender(1)	-.012	.353	.001	1	.972	.988	.495	1.971
Duration in China	-.331	.139	5.632	1	.018*	.718	.547	.944
Chinese proficiency	.390	.200	3.793	1	.051	1.477	.998	2.186
English proficiency	.074	.253	.086	1	.769	1.077	.656	1.769
Avoidance	-.324	.174	3.474	1	.062	.723	.514	1.017
Anxiety	-.206	.226	.833	1	.361	.814	.523	1.266
Host ties	.426	.246	2.991	1	.084	1.531	.945	2.480
International ties	-.145	.226	.412	1	.521	.865	.555	1.347
Co-national ties	.213	.260	.669	1	.413	1.237	.743	2.060
constant	1.577	2.001	.622	1	.430	4.842		

*P < .05,

**P < .01,

***P < .005.

### Predictors of assimilation

A binary logistic regression analysis was used to predict the chance that the international student would adopt assimilation. The Hosmer and Lemeshow test showed that the full model was valuable, with χ2 = 5.017, df = 8,*P*>0.05. The overall correct percentage was 86%. The full model explained 14.0% (Nagelkerke’s R^2^) of variance in the assimilation attitude of international students, with host ties as the significant variable. As shown in Table 4, host ties positively influence assimilation attitude of international students. The odds ratio for host ties was 2.412, indicating that holding all other variables constant in the model, with a one point increase on the 4-point social support Scale, the possibility that the participants adopted the assimilation attitude was 2.412 times.

**Table 4:** Logistic regression predicting likelihood of adopting assimilation attitude.

	B	S.E	Wald	df	P	Exp(B)	95% CI for OR	
							lower	upper
Gender(1)	-.346	.429	.650	1	.420	.707	.305	1.641
Duration in China	.199	.145	1.888	1	.169	1.220	.919	1.621
Chinese proficiency	-.129	.248	.272	1	.602	.879	.540	1.429
English proficiency	.336	.291	1.329	1	.249	1.399	.791	2.474
Avoidance	-.191	.202	.890	1	.346	.826	.556	1.228
Anxiety	.529	.294	3.251	1	.071	1.698	.955	3.019
Host ties	.880	.303	8.425	1	.004*	2.412	1.331	4.371
International ties	-.142	.280	.257	1	.612	.868	.501	1.502
Co-national ties	-.486	.346	1.969	1	.161	.615	.312	1.213
constant	-2.059	2.278	.817	1	.366	.128		

*P < .05,

**P < .01,

***P < .005.

### Predictors of integration

A binary logistic regression analysis was used to predict the chance that an international student would adopt integration. The Hosmer and Lemeshow test showed that the full model was valuable, with χ2 = 4.668, df = 8, *P*>0.05. The overall correct percentage was 86%. The full model explained 30.5% (Nagelkerke’s R^2^) of variance in the integration attitude of international students, with Chinese proficiency, avoidance and host friends as the significant variables. As shown in Table 5, Chinese proficiency and host ties positively influence integration attitude of international students, avoidance negatively influence integration attitude of international students. The odds ratio for Chinese proficiency, avoidance and host ties was 1.751,0.672 and 3.081, respectively, indicating that holding all other variables constant in the model, with a one point increase on 5-point Chinese proficiency, 7-point attachment Scale and 4-point social support Scale, the possibility that the participants employed the integration attitude was 1.751,0.672 and 3.081 times, respectively.

**Table 5:** Logistic regression predicting likelihood of adopting integration attitude.

	B	S.E	Wald	df	P	Exp(B)	95% CI for OR	
							lower	upper
Gender(1)	-.414	.312	1.755	1	.185	.661	.358	1.220
Duration in China	.058	.114	.257	1	.612	1.060	.847	1.325
Chinese proficiency	.560	.198	7.969	1	.005*	1.751	1.187	2.583
English proficiency	-.270	.237	1.307	1	.253	.763	.480	1.213
Avoidance	-.397	.151	6.887	1	.009*	.672	.500	.904
Anxiety	.226	.214	1.119	1	.290	1.254	.825	1.906
Host ties	1.125	.234	23.126	1	.000*	3.081	1.948	4.875
International ties	-.056	.205	.076	1	.783	.945	.632	1.413
Co-national ties	.068	.242	.079	1	.778	1.071	.666	1.720
constant	-3.176	1.837	2.990	1	.084	.042		

*P < .05,

**P < .01,

***P < .005.

### Predictors of marginalization

A binary logistic regression analysis was used to predict the chance that the international student would adopt marginalization. The Hosmer and Lemeshow test showed that the full model was valuable, with χ2 = 7.349, df = 8, *P*>0.05. The overall correct percentage was 90.4%. The full model explained 30.6% (Nagelkerke’s R^2^) of variance in the marginalization attitude of international students, with duration in China, Chinese proficiency, English proficiency and host friends as the significant variables. As shown in Table 6, English proficiency positively influence marginalization attitude of international students, duration in China, Chinese proficiency and host ties negatively influence marginalization attitude of international students. The odds ratio for duration in China, Chinese proficiency, English proficiency and host ties was 0.658,0.543,4.422 and 0.460, respectively, indicating that holding all other variables constant in the model, with a one point increase on those Scales, the possibility that the participants employed the marginalization attitude was 0.658,0.543,4.422 and 0.460 times, respectively.

**Table 6:** Logistic regression predicting likelihood of adopting marginalization attitude.

	B	S.E	Wald	df	P	Exp(B)	95% CI for OR	
							lower	upper
Gender(1)	.577	.546	1.117	1	.290	1.781	.611	5.192
Duration in China	-.418	.179	5.432	1	.020*	.658	.463	.936
Chinese proficiency	-.610	.277	4.837	1	.028*	.543	.315	.936
English proficiency	1.487	.573	6.725	1	.010*	4.422	1.438	13.602
Avoidance	.075	.239	.099	1	.754	1.078	.675	1.723
Anxiety	-.340	.345	.972	1	.324	.712	.362	1.400
Host ties	-.777	.344	5.106	1	.024*	.460	.234	.902
International ties	-.340	.367	.857	1	.354	.712	.347	1.461
Co-national ties	-.280	.415	.456	1	.499	.756	.335	1.703
constant	-1.774	3.592	.244	1	.622	.170		

*P < .05,

**P < .01,

***P < .005.

## Discussion

This study sought to illustrate the influence of several predictors such as demographic factors, attachment style, media usage, and social support on the four acculturation strategies, and found out what specific factors could impact integration, assimilation, separation, and marginalization strategies of international students in China. In line with predictions derived from acculturation mode, findings indicated that duration in China, Chinese proficiency, English proficiency, host social ties and avoidance attachment style could predict the acculturation attitude of international students in China.

### The most prevalent acculturation attitude

Results revealed that the most prevalent acculturation strategy for the international students in China is integration, which was in accordance with previous researches [32, 39]. Two explanations may account for this result. Firstly, China is a country with 56 ethnic groups, each ethnic group has its own culture, so, China is a multicultural country, the more culturally diverse the host society is, the more inclusive it is in terms of allowing for foreigners to keep their own culture while learning host culture, so integration would be the most beneficial acculturation attitude [40]. Secondly, most of participants come from countries which are on good terms or in bilateral relationship with China, such as Pakistan, Zimbabwe, Ethiopia, Tanzania. They had a positive image of Chinese as tolerant, kind, and desire for contact, in turn, this positive stereotype was associated with a more pleasant attitude towards international students as a whole, which is also witnessed by other scholars [41]. Lastly, a lot of cultural activities were being held at the university, it was a good opportunity to show and understand different culture, It can be understood that the shorter cultural distance between the mainstream and heritage culture, the higher the tendency by international students to adapt integration attitude.

### The influence factors of acculturation attitude

Consistent with previous studies [30, 34], duration of stay in China is found to predict less usage of separation and marginalization attitude. Since separation and marginalization attitude is concerned with rejection the local mainstream culture, the factors which heighten opportunity of contacting host culture in international students are negatively related to separation and marginalization attitude. Emiko S. Kashima and Evelyn Loh [33] founded the positive correlation between duration of host country and host cultural knowledge. The longer duration of China, the more chance it is for the international students to interact with host people and learn host culture, which can discourage separation and marginalization attitude.

Contrary to previous studies [5, 35], this study found that higher level of English proficiency and/or lower level of Chinese proficiency predicted the preference of marginalization attitude. The main explanation of this is that most of participants in our study come from countries which English or Chinese is not official or mother language. Chinese is official and host language in China and it is the main communication tool for international students at academic and social settings. A Lower level of host language may hinder interaction with the host society [42], but the limited host language proficiency might be compensated by interaction with the foreigner(exclude Chinese) since English is the lingua franca among international students, so English proficiency is negatively with heritage orientation [5]. Moreover, we founded that higher level of Chinese language proficiency predicted the preference of integration attitude. strong command of host language increases the ability of foreigner to understand local norms, values and culture and communicate with host nationals [4, 5], which can encourage integration attitude.

The current findings provide clear support for the hypothesis that host ties are positively and significantly associate with assimilation and integration attitude, but negatively associate with marginalization attitude. Host-national contact were positively associated with multiculturalism [43]. Through host nationals contacts, international students are able to learn and understand norms, rules and behaviors of local people, thus previously unexplained behavior can be interpreted more readily now [19]. International students who had more contact with locals were likely to have more positive feelings about the host culture [44]. Host ties play a paramount role in the mainstream culture identification which is positively associated with assimilation and integration attitude, but negatively associate with marginalization attitude. Unlike previous studies [20, 45, 46], this study found that conational ties and international ties are no connection with acculturation attitude, two explanations may account for this result. In our study, some of participants have few compatriots to form strong conational ties in our school, in addition, most of them are on short term learning, the sojourn of international students in China t may impact the degree to which international students are motivated to formed new relations, the intention to stay in the host country of international students after graduation can impel their willingness to learn the culture of the host country, which in turn influences the quality and quantity of social ties [32].

Avoidance attachment was found to have a negative association with integration attitude. According to the internal working model which consists of mental representations of oneself, others, and relationships [47], if the attachment figure is perceived as consistent rejection or unavailability, negative other-representation were developed. The higher the participants scored on the avoidance attachment characterized by fear of interpersonal dependence, distrust of others, the more they tended to avoid contact with locals [26], leading to a negativeassociated with integration. In contrast to other studies in the American and Netherlands [26, 27, 48], the findings about association attachment style and acculturation attitude in our study are different, one reasoning is that different attachment scale were used.

Our study is the first to investigate predictors of acculturation attitude from attachment style and social ties aspect with data collected from an international student in China, which can provide valuable information for education policymakers in China.

In spite of its insights into existing acculturation research, several limitations should be noted. Firstly, Although Yangtze University is one of the famous universities with the largest number of nationalities and participants were selected by multistage sampling method to ensure the convenience of sampling and the representativeness of samples at the same time, the sample was selected from only one university, so the conclusion may have some limitations. Despite this limitation, this research is only a pilot study, future studies with large and more heterogeneous samples are required to gain a better understanding of current findings.

Secondly, the data collected for this study by cross-sectional study which has an inherent weakness in identifying causality. Previous researches found that acculturation attitudes and social ties change over time, although findings are inconclusive as to how these changes might come about. In the recent research about acculturation attitudes, Juan Serrano-S´anchez and colleague [49], who followed students whom registered for international exchange programs in Germany, found that host-oriented attitudes decreased across the transition from 2 weeks before departure to 8 weeks after the transition abroad and stayed rather stable from 8 weeks to 28 weeks after the transition abroad, while the changes of the home-oriented attitudes were totally in the opposite direction. In contrast, in Rupert Brown’s longitudinal study with immigrants in British, desire for cultural maintenance and inter group contact was increased as time went [14]. On the social ties aspect, a more recent longitudinal study with international students in Argentina, show that co-nationals and muti-natioanls friendship were stronger than host nationals friendship upon arrival. However, both host national and muti-national friendships increased over time [50]. In contrast, Rienties and Nolan [51] conducted a study of international students in the United Kingdom and found that different cultural backgrounds students develop different friendships over time, as Asian students became more separated from local students over time, while other international students integrated well into co-national and host national groups. Overall, additional longitudinal studies are needed to understand how social ties and acculturation attitude develop and change over time and verify the proposed relationship. In addition, previous studies found that international students who have secure adult attachment style are extroverts which can establish all kinds of social ties in the new environment faster [52]. The strength of the links between those social ties will affect the acculturation attitude, therefore, whether adult attachment style can affect the acculturation attitude through the social ties remains to be studied.

## Supporting information

S1 File Constent information. (PDF)

S2 File Ethical inspection. (PDF)

S3 File Dataset. (SAV)
