# Supplementary material for: Predictors of acculturation attitude of international students in China
Source: PLoS One. 2021 Nov 30;16(11):e0260616. doi: 10.1371/journal.pone.0260616 (PMC8631655; doi:10.1371/journal.pone.0260616)
Supplement: S1 File — (PDF) [file pone.0260616.s001.pdf]

A survey about cross cultural adaption of international students in China

Dear international students,

I am a teacher of Yangtze university. I am doing a survey about cross cultural adaption of international students in China. The purpose of my study is to help international student adapt daily life efficiently in China. ***The research received approval by Medical ethics committee of Yangtze university. If you voluntarily participate in this research project, and agree to use those data for the publication of this study, please finish the questionnaire. When you complete and submit the questionnaire, it means I received written consent from you and the consent is informed.*** Your participation is very important for us and your answer is anonymous and strictly confidential. ***There are no right or wrong answers. However, you must answer each statement which is given below as honestly as possible.*** Thank you very much. Best wishes for your life in China. (Please make √ in appropriate answers.)

Part one

1. Gender
   ① Male ②Female
2. Age: ________
3. Nationality
   ① Asia ②Africa ③ Europe ④America ⑤ Oceania
4. Marital status is ________
   ① Unmarried ② Married
5. Your religious belief is ________
   ① Christian ② Islam ③ Buddhism ④ Other
6. How long have you lived in China?
   ① 1-12months ② 13-24months ③ 25-36months ④ over 36 months
7. How good do you think your overall Chinese language ability is? ________
   ① very poor ②poor ③ moderate ④good ⑤ very good
8. How good do you think your overall English language ability is? ________
   ① very poor ②poor ③ moderate ④good ⑤ very good
9. Have you ever been abroad before you came to China?
   ① Never ②Yes
10. Before coming to China, I know ________ about China.
    ① very little ② little ③ moderate ④ much ⑤ very much

Part two

Social ties scale

Below are some questions about the kind of help and support you have available to you in coping with your life at present. The questions refer to three different groups of people who might have been providing support to you IN TEE LAST MONTH. For each item, please circle the alternative which shows your answer.
