# Supplementary material for: Predictors of acculturation attitude of international students in China
Source: PLoS One. 2021 Nov 30;16(11):e0260616. doi: 10.1371/journal.pone.0260616 (PMC8631655; doi:10.1371/journal.pone.0260616)
Supplement: S2 File — (PDF) [file pone.0260616.s002.pdf]

The Tab of Ethical Inspection

Some information about the applicant and study	Applicant institute：Yangtze university
	Academic title：Lecturer
	Study title: Cross sectional study of international students acculturation statues and its relationship with adult attachment in China.
	Study aim：Objective to investigate acculturation statues of international students in China and the influence of adult attachment and social ties on acculturation statues.
	Signature of applicant: Man Luo<br>Phone：15027075640
Inspection contents	Does this project obtain informed consent from all participant?<br><br>Written consent was obtained from the participants and the consent was informed. All the participants were over 18 years old.
Results of inspection	Director of Ethics committee:<br><br>The project fully protect the rights and interests of subjects, conform to the requirements of the medical ethics committee. Agreed to research plan.<br><br>Medical ethics committee (seal) :<br>江大学医学院 伦理委员会<br><br>Date September 16th, 2019
